# Molecular epidemiology of human papillomavirus among HIV infected women in developing countries: systematic review and meta-analysis

**DOI:** 10.1186/s12985-020-01448-1

**Published:** 2020-11-16

**Authors:** Agajie Likie Bogale, Nega Berhe Belay, Girmay Medhin, Jemal Haidar Ali

**Affiliations:** 1 Ethiopian Public Health Institute, and Addis Ababa University, P.O. Box 1242, Addis Ababa, Ethiopia; 2 Department of Tropical and Infectious Diseases, Aklilu Lemma Institute of Pathobiology, Addis Ababa University, P.O. Box 1176, Addis Ababa, Ethiopia; 3 School of Public Health, College of Health Sciences, Addis Ababa University, 1000, P.O. Box 27285, Addis Ababa, Ethiopia

**Keywords:** Human papillomavirus, Human immunodeficiency virus, Genotype, Women/females, Meta-analysis, Developing countries

## Abstract

**Background:**

Although, there is a variable burden of human papillomavirus (HPV) in women infected with HIV in developing countries, there are few studies that attempted to surmise such variable evidences. This review aimed to estimate the pooled prevalence of HPV genotype distribution and risk factors contributing to HPV infection among women infected with HIV in low- and middle-income countries.

**Methods:**

We conducted a systematic review and meta-analysis of studies conducted in developing countries and reported HPV prevalence. We searched electronic databases: PubMed/Medline, SCOPUS, ScienceDirect, Excerpta Medical Database from Elsevier, Web of science, Cumulative Index of Nursing and allied Health Sciences and Google scholar databases to retrieve primary studies published in English language till 11th August 2019. We used random-effects model to estimate the pooled prevalence of HPV genotypes, and funnel plot to assess publication bias. The registration number of this review study protocol is CRD42019123549.

**Results:**

We included nineteen studies with a total of 8,175 participants in this review. The prevalence of HPV was extremely heterogeneous across the studies (χ^2^_=_ 3782.80, *p* value < 0.001, I^2^ = 99.6%). The estimated pooled prevalence of all HPV genotypes was 63.0% (95% CI: 48.0–78.0) while the pooled prevalence of high risk and low risk HPV genotypes were 51.0% (95% CI: 38.0–63.0) and 28.0% (95% CI: 12.0–43.0), respectively. The pooled prevalence of HPV genotype 16 was 20%, while genotype 18 and 52 were 15% and 13%, respectively. Different risk factors reported for HPV infection and the frequently reported were low CD4 count below 200 cells/mm^3^ and high HIV viral load.

**Conclusion:**

The pooled prevalence of HPV among HIV infected women in low- and middle-income countries was considerable and the proportion of high risk HPV genotypes were high when compared with low risk genotypes. Therefore, it is essential for the HPV prevention program to prevent the double burden of HPV and HIV in women.

## Background

The papillomavirus is a heterogeneous group of DNA virus with circular, non-enveloped, double-stranded DNA genomes [1, 2]. This virus infects humans and different species of animals [2]. The virus is discovered from the horn of Cottontail rabbit at the beginning of 1930s [3] and also revealed as a main cause of human cervical cancer in 1970s [4]. More than 300 papillomaviruses have been identified and completely sequenced, including over 200 human papillomaviruses [5]. The high-risk carcinogenic types of HPV currently designated by the International Agency for Research on Cancer (IARC) are HPV16, HPV18, HPV31, HPV33, HPV35, HPV39, HPV45, HPV51, HPV52, HPV56, HPV58, and HPV59. The HPV68 is classified as probably carcinogenic, and HPV26, HPV30, HPV34, HPV53, HPV66, HPV67, HPV 69, HPV70, HPV73, HPV82, HPV85, and HPV97 have been associated with rare cases of cervical cancer and are considered probable carcinogens [6, 7]. Genotype 6 and 11 are low-risk types that cause genital and skin warts [8]. Genital HPV infections are very common and prevalent in the age range of 18 to 30 years [9, 10]. Infection of the cervix with HPV is necessary to cause cervical neoplasia and cervical cancer [11, 12], and integration of viral DNA into the host genome is necessary for persistent infection which could lead to the development of cervical dysplasia [11].

The prevalence of HPV is variable across the world. The study reported from developed countries indicate that the prevalence of HPV was 11 to 12% [13]. The recent global estimate indicates 11.7% of the HPV infection burden in the world [14]. The occurrence of about 85% of infected cases and 88% of the deaths due to cervical cancer is in developing countries [11].

The highest prevalence was reported in sub-Saharan Africa (24%), Eastern Europe (21%) and Latin America (16%) [15].

The burden of HPV infection is higher in HIV infected women (50.8%) than un-infected (22.6%) [16] and 78.8% among HIV infected than 34.4% of un-infected women [17]. Similarly, high-risk oncogenic HPV types is higher among HIV infected than un-infected women (48.4% vs. 17.3%) [16]. Other studies reported a prevalence of 68.0% [18] and 33.2% [19]. Moreover, the study reported from developing countries indicated extremely variable prevalence of HPV that ranges from 20 to 70% [20]. The prevalence of low-risk HPV types were 3.6 to 5.6 times higher in HIV-sero-positive women when compare to HIV sero-negative's [8].

Several risk factors are reported to be associated with HPV infection and these include HIV infection, other STIs (e.g., chlamydia, herpes simplex virus), and multiple sexual partners [11, 21]. There are also other factors that mediate HPV infection such as cigarette smoking, oral contraceptive or hormonal contraceptive use, chronic inflammation and immunosuppressive conditions [10, 11, 21, 22]. Dietary factors, socioeconomic status, race/ethnicity, geographic disparity and polymorphisms in the human leukocyte antigen system are additional factors that could mediate HPV infection [10, 11, 21, 22]. Being young age and having active sexual behavior are key risk factors for HPV acquisition and persistence of the infection [22].

HIV infection increases the risk of cervical infection due to high-risk HPV genotypes that induces high-grade cervical squamous intraepithelial lesions (HSILs), which in turn leads to the development of pre-invasive cervical lesions and invasive cervical cancer (ICC) [23–25]. HIV infection could alter the natural history of HPV infection through decreasing the self-clearance rate of infection and increasing progression to high grade and invasive lesions [24]. Furthermore, the incidence of HPV infection is three times higher in HIV-positive women [25], and can cause cervical cancer than their counterparts [26]. Nonetheless, with the exception of the systematic review and meta-analysis done in Kenya [27], evidences in this regard showing the burden and molecular distribution of HPV in low and middle income countries (LMICs) is limited [28]. Therefore, this review aims to fill the identified gaps by estimating the pooled prevalence of HPV, and investigating the factors associated with HPV infection among HIV infected women in LMICs.

## Methods

### Search strategy and screening of papers

We conducted a systematic review and meta-analysis of published articles to estimate the pooled prevalence of HPV in LMICs. We systematically searched the papers published in the following electronic databases; PubMed/MEDLINE, SCOPUS, Science Direct, Excerpta Medical Database from Elsevier (EMBASE), Web of science, Cumulative Index of Nursing and Allied Health Sciences (CINAHL) and Google scholar. The review was conducted in accordance with Preferred Reporting Items for Systematic Reviews and Meta-Analyses (PRISMA) standard [29]. We used a search strategy by combining the following key terms: molecular, molecular epidemiology, human papillomavirus, or HPV, papillomavaridae, Human immunodeficiency virus (HIV), AIDS (acquired human immunodeficiency syndrome), HIV infected, HIV positive, HIV sero-reactive, women, female and girl. We used Truncation(*) to manage spelling variation during search: infect* or positive, wom*n or female* or girl*. We used both free text and Medical subject heading [MeSH] terms during electronic database search.

PubMed database search strategy was:((((molecular[tiab] OR "Molecular Epidemiology"[Mesh]) AND ((Human papillomavirus[tiab] OR HPV[tiab]) OR "papillomaviridae"[MeSH Terms])) AND (((Human immunodeficiency virus[tiab] OR HIV[tiab]) OR "hiv"[MeSH Terms]) OR "hiv"[MeSH Terms])) AND (infected[tiab] OR positive[tiab])) AND (((women[tiab] OR females[tiab]) OR "women"[MeSH Terms]) OR "female"[MeSH Terms]) AND (("1966/01/01"[PDAT]: "2019/08/11"[PDAT]) AND "humans"[MeSH Terms] AND English[lang] AND "female"[MeSH Terms]).

The search was repeated to identify the consistency of search terms and results. Two authors independently reviewed the titles, abstracts and full articles of the retrieved studies.

### Study inclusion and exclusion criteria

**Fig. 1:**
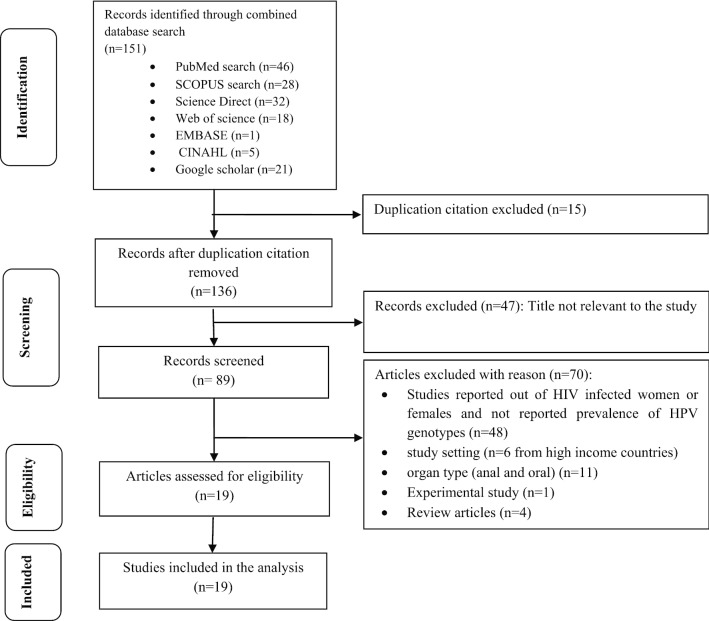
Flow diagram of studies reviewed, screened and included

We included a cross sectional and cohort studies conducted in LMICs based on World Bank Country Classifications, 2018 [30] and that reported prevalence of HPV genotypes. The inclusion was restricted to the papers published in English language without limiting publication year till 11th August 2019. We excluded studies that did not clearly state the study design, outcome measured, the study conducted on HIV negative women alone, conducted in high-income countries, and the study reported HPV genotype from anal and oral organ types (Fig. 1).

### Study quality assessment

We assessed the quality of included studies by using the 14 items Quality Assessment Tool for Observational Cohort and Cross-Sectional studies NHLBI, NIH [31]. This assessment tool mainly focused on research question, study population, eligibility criteria (inclusion and exclusion criteria of study participants), sample size justification, exposure measures and assessment, sufficient time frame to see an effect, outcome measures and blinding of outcome assessors, follow up rate, and statistical analysis. The quality assessment was rated as good, fair and poor based on the quality assessment tool criteria. The maximum score indicating high quality was 14 and the lowest possible score was zero. The rating values of the included studies in terms of their quality were based on their design. Cross-sectional type do not consider the items which fit for cohort and taken as not-applicable (NA) and thus, the rating values were not taken from the possible maximum score (i.e. 14). In this review, all scores are written in percentage.

### Data extraction

Data from eligible abstract and/or full text of the articles were extracted by considering the outcome variables (i.e. prevalence or proportion of HPV genotypes, magnitude of cancer causing HPVs or high risk (HR) HPV genotypes and low-risk HPV types), and factors that could potentially be associated with these outcomes. The characteristics of study participants of an eligible paper such as age range, mean or median age, sex, HIV sero-status, the prevalence of HPV genotype were also extracted. Study characteristics such as first author, year of publication, study duration, study setting, study location or country, study design, sample size were also extracted (Table 1). Other extracted data include the prevalence of different HPV genotypes (Table 2), factors which could potentially be associated with HPV infection and diagnostic methods applied to detect HPV infection (Table 3).

**Table 1:** Characteristics of included studies to estimate the pooled effect of HPV among HIV-infected women in LMICs

First author	Year	Study setting	Study location	Continent	Study design	Sample size	HPV prevalence	HR HPV prevalence	LR HPV prevalence	Age category in years and proportion of HPV	Mean age in years	Median age in years	Age range in years
Veldhuijzen et al. [36]	2011	Health facility	Rwanda	Africa	Cross sectional and cohort	192	139	98	105	< 30 years = 56%, ≥ 30 years = 43.1%		27	≥ 18
Sinayobye et al. [37]	2014	District Hospital	Rwanda	Africa	Cross sectional	1228		390		30–34 = 46.8%, 35–39 = 27.9%, 40–44 = 28.1%, 45–49 = 25.7%, 50–61 = 26.4%	40		30–60
Rocha-Brischiliari et al. [38]	2014	Health Facility	Brazil	South America	Standardized questionnaireaand medical record review	178	83	57	26	18–30 = 60.7%, 31–40 = 43.3%, > 40 = 44.4%	–	–	18–66
Bollen et al. [43]	2006	Bangkok hospitals	Thailand	Asia	Medical records review	256	91	60		< 20 = 34.5%, 20–25 = 41.2%, 25–30 = 32.1%, > 30 = 25.7%		25	17–39
McDonald et al. [44]	2014	clinic site	South Africa	Africa	Cohort	1371	718			17–29 = 56.5%, 30–39 = 53.6%, 40–65 = 33.3%		34	17–65
Firnhaber et al. [45]	2010	teaching hospital	South Africa	Africa	Cross-sectional	1010	191					34	18–65
Firnhaber et al. [46]	2009	teaching hospital	South Africa	Africa	Cohort	148	141	123			36		18–65
Nweke et al. [41]	2013	Gynecologic outpatient clinic	Nigeria	Africa	Cross-sectional	98	45	37			36.8		≥ 18
Denny et al. [47]	2008	Primary health care clinic and colposcopy clinic	South Africa	Africa	Longitudinal cohort study	400		269			29.1		18–54
Akarolo-Anthony et al. [42]	2013	Hospital	Nigeria	Africa	Cross-sectional	149		53			36.6		≥ 18
Sahasrabuddhe et al. [48]	2007	University Teaching Hospita	Zambia	Africa	Cross sectional	145	141	131	87		36.2		
Rousseau et al. [49]	2006	public health facility	Burkina Faso	Africa	Cross-sectional	126	110	89	21			28	16–54
Helen [52]	2017	HIV treatment centers	Burkina Faso and South Africa	Africa	Prospective cohort	1238	1151	842	109			35	≥ 15
Hawes et al. [23]	2003	Infectious-disease clinic	Senegal	Africa	Colposcopically directed cervical biopsy specimens	426	289	222			33.6		> 15
Mattos et al. [39]	2011	(STI/AIDS) clinic	Vitoria, Brazil	South America	Descriptive study	112	33	18	15			29	14–51
Nicol et al. [40]	2013	Institute of clinical research, Hospital and HIV VCT	Brazil	South America	Cross sectional	532	369				37.7		
Sagna et al. [35]	2010	Medical center	Burkina Faso	Africa		156	91				33.65		19–45
Munoz et al. [51]	2013	Health facility	Colombia	South America	Cross sectional	194	136			20–34 years = 65 (73.9%), 35–49 years = 42 (60%), ≥ 50 years = 29(80.6%)	38		
Camargo et al. [50]	2014	Hospital based	Colombia	South America	Cross-sectional	216	149				37.5		20–73

**Table 2:** Prevalence of different HPV genotypes included in the meta-analysis of women infected with HIV in LMICs

References	Year	HPV 52	HPV 58	HPV 51	HPV 16	HPV 45	HPV 35	HPV 18	HPV 31	HPV 66	HPV 59	HPV 82	HPV 56	HPV 39	HPV 53	HPV 33	HPV 68	HPV 69	HPV 73	HPV 26	HPV 67	HPV 70
Veldhuijzen et al. [36]	2011	27	21	21	15	15	15															
Sinayobye et al. [37]	2014																					
Rocha-Brischiliari et al. [38]	2014		6	11	11	1		4	11	6	5	5	4	1	3	3	1		3			
Bollen et al. [43]	2006	12	8	10	11	4	3	10	1	4	1		5	14	11							
McDonald et al. [44]	2014	74	108	70	112	78	117	85	56		45		51	51		59	85					
Firnhaber et al. [45]	2010																					
Firnhaber et al. [46]	2009	20	14	20	45	25	30	27	11	18	16	10	22	14	29	12	12	12	12	4	2	
Nweke et al. [41]	2013	15					7		16						9							
Denny et al. [47]	2008	60	39	35	60	34	57	44	24	36	32	21	35	29	59	19	34	12	22	15	8	41
Akarolo-Anthony et al. [42]	2013	3	10	4	5	7	13	5	3		8		11	4		6	5					
Sahasrabuddhe et al. [48]	2007	65	35	22	25	25	25	19	21	21	12	7	18	18	30	12	20		10	9		
Rousseau et al. [49]	2006	19	12	11	10		12	8	9						8							
Helen [52]	2017																					
Hawes et al. [23]	2003																					
Mattos et al. [39]	2011																					
Nicol et al. [40]	2013				299			202														
Sagna et al. [35]	2010				9			33														
Munoz et al. [51]	2013		39		104	15		59	64							40						
Camargo et al. [50]	2014		44		100	19		66	71							40						

The number in the table indicates the prevalence of different HPV genotypes included in the study. The proportion reported in the studies converted to number by multiplying the total sample size of each study by the proportion in percent for each required variables. This is very easy to run metaprop command in STATA software. Preparing data for meta-analysis in suitable form is the first step in quick work flow of analysis

**Table 3:** Molecular genotyping techniques and associated factors for HPV infection

References	Year of publication	Molecular technique used for genotyping	Associated factors	Quality assessment score
Veldhuijzen et al. [36]	2011	Linear Array HPV Genotyping Test (LA)		84.6%
Sinayobye et al. [37]	2014	careHPV	Lower CD4 count < 200, history of 3 or more sexual partners, and history of using hormonal contraception	87.5%
Rocha-Brischiliari et al. [38]	2014	Genotyping using PCR-restriction fragment length polymorphism analysis	Hormonal contraceptive use and current smoker	100%
Bollen et al. [43]	2006	Reverse line-blot hybridization	Higher HIV-plasma viral load	87.5%
McDonald et al. [44]	2014	Prototype polymerase chain reaction (PCR)-based line blot assay and PCR-based, LinearArrayHPVTypingAssay		83.3%
Firnhaber et al. [45]	2010	Linear Array genotyping assay (Roche)		87.5%
Firnhaber et al. [46]	2009	Roche Linear Array HPV genotyping test		91.7%
Nweke et al. [41]	2013	HPV GenoArray test kits		75.0%
Denny et al. [47]	2008	Roche Linear Array HPV genotyping test	Low CD4 count and high viral load	83.3%
Akarolo-Anthony et al. [42]	2013	Roche Linear Array HPV Genotyping Test		87.5%
Sahasrabuddhe et al. [48]	2007	Roche Linear Arrays HPV genotyping test	HRHPV associated with low CD4 count < 200	75.0%
Rousseau et al. [49]	2006	INNO-LiPA HPV Genotyping v2 test	High prevalence of HPV on HIV infection	87.5%
Helen [52]	2017	INNO-LiPA HPV genotyping Extra® assay	Injectable contraceptive and VL > 1000	91.7%
Hawes et al. [23]	2003	PCR -based molecular tests	High VL and low CD4 count	87.5%
Mattos et al. [39]	2011	Restriction Fragment Length Polymorphism		75.0%
Nicol et al. [40]	2013	VLPs-based ELISA		62.5%
Sagna et al. [35]	2010	PCR -based molecular tests		Only abstract
Munoz et al. [51]	2013	PCR-based molecular tests		87.5%
Camargo et al. [50]	2014	PCR-based molecular tests	CD4 < 500 and high VL have association with HPV detection	100%

HRHPV, high risk human papilloma virus; HIV, human immunodeficiency virus; VL, viral load; VLP, virus like particles; PCR, polymerase chain reaction; LiPA, line probe assay; ELISA, enzyme linked immuno-sorbant assay; CD4, cluster of differentiation 4

The majority of the studies included in our review had more than eighty percent and the lowest score observed was 62.5% in terms of quality. There was however one abstract included in the review, which was difficult to assess the quality of the article (Table 3).

### Statistical analysis

We estimated the pooled prevalence of HPV with its 95% Confidence Interval (CI) using random effects meta-analysis model assuming the true effect size varies between studies [32]. The proportion of HPV reported in each study is multiplied by its sample size to express patients with HPV infection in number, and data presented in forest plot. Heterogeneity in the prevalence of different studies was assessed using Chi-square (χ^2^) based Q test with significant level of *p* value < 0.1 and I^2^. The I^2^ value of25% indicates low heterogeneity while 50% moderate and 75% high [33]. The potential publication bias was assessed using a funnel plot. If the 95% of the point estimate of studies lie within the funnel plot defined by straight lines, then that indicates the absence of heterogeneity [34]. The potential sources of heterogeneity were assessed by doing subgroup analysis and moment based meta regression. Meta-regression extends subgroup analyses and allows to estimate effect size. Data analysis was conducted using STATA version 14.

## Results

### Study characteristics

We included 19 studies in our review (Fig. 1). These studies were reported from Rwanda [36, 37], Brazil [38–40], Nigeria [41, 42] Thailand [43], South Africa [44–47], Zambia [48], Burkinafaso [35, 49], Senegal [23] and Colombia [50, 51]. There was one study conducted in two countries Burkinafaso and South Africa [52]. Five studies were from South America (three from Brazil and two from Colombia), one study from Asia (Thailand) and the rest were from African countries. All of the studies were from health facilities (Hospital and clinic) and the majority were cross sectional studies. The publication year varied from 2003 to 2017 while the majorities (13 articles) were published after 2009. Eight studies were published in 2013 and 2014. The maximum sample size was 1371 [44] and the minimum was 98 [41]. The age of participants ranged from 14 to 73 years [39, 50]. Three studies didn't mention the upper age range of the participants [23, 36, 42] (Table 1).

### Pooled prevalence of HPV

**Fig. 2:**
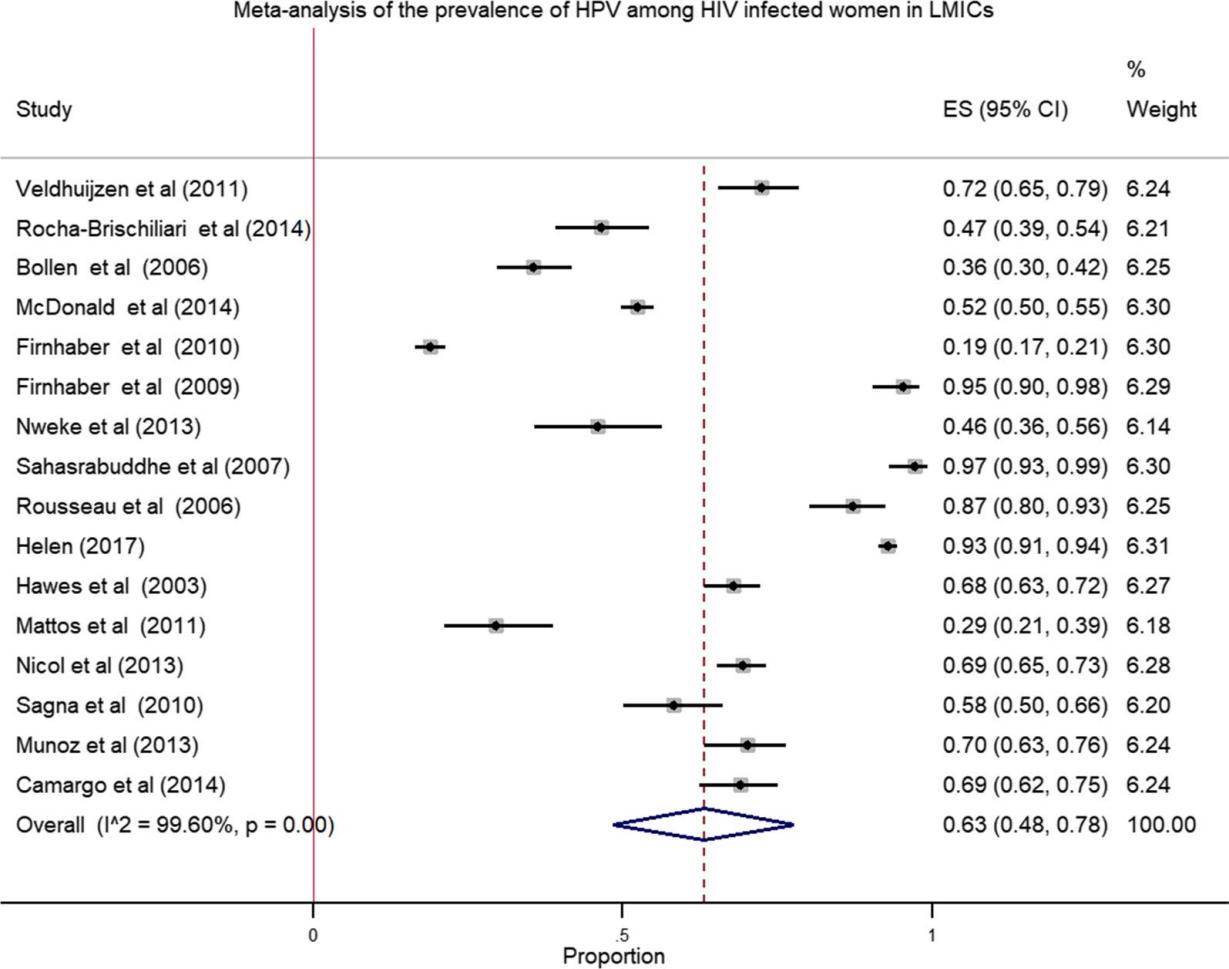
Forest plot to estimate the pooled prevalence of human papillomavirus infection among HIV infected women with 95% CI (the estimate weighted based on random effects model): ES-Effect Size equivalent to prevalence, CI—confidence interval. In the plot, the diamond shows the pooled result and the boxes show the effect estimates from the single studies. The purple dotted vertical line indicates pooled estimate. The purple solid vertical line indicates the reference line at zero indicating no effect. The horizontal line through the boxes illustrate the length of the confidence interval and the boxes show the effect estimates from the single studies

**Fig. 3:**
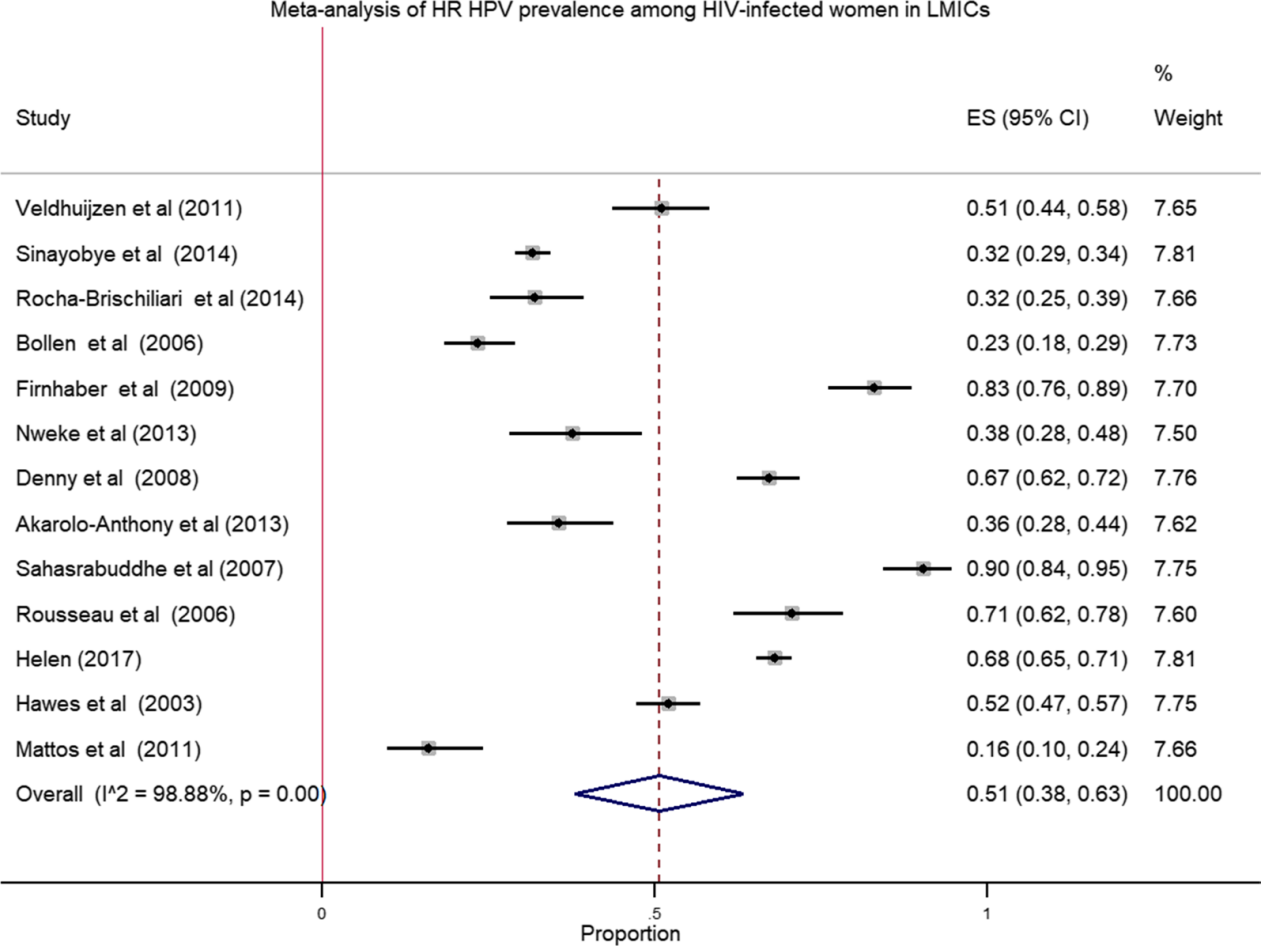
Forest plot to estimate the pooled prevalence of high risk human papillomavirus infection among HIV infected women (the estimate weighted based on random effects model): ES—effect Size equivalent to prevalence, CI—confidence interval. In the plot, the diamond shows the pooled result and the boxes show the effect estimates from the single studies. The purple dotted vertical line indicates pooled estimate. The purple solid vertical line indicates the reference line at zero indicating no effect. The horizontal line through the boxes illustrate the length of the confidence interval and the boxes show the effect estimates from the single studies

**Fig. 4:**
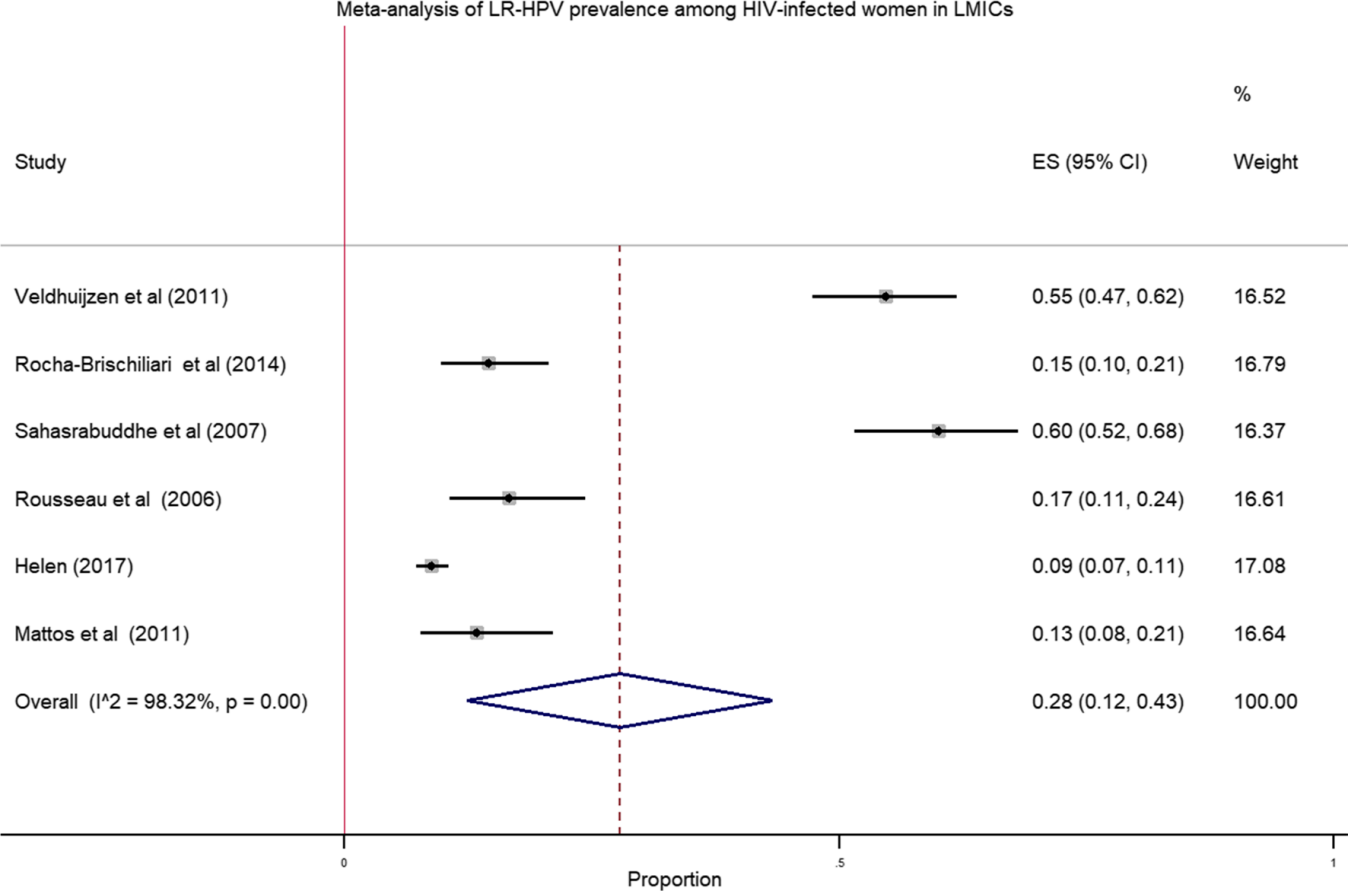
Forest plot to estimate the pooled prevalence of low risk human papillomavirus infection among HIV infected women with 95% CI (the estimate weighted based on random effects model): ES—effect size equivalent to prevalence, CI—confidence interval. In the plot, the diamond shows the pooled result and the boxes show the effect estimates from the single studies. The purple dotted vertical line indicates pooled estimate. The purple solid vertical line indicates the reference line at zero indicating no effect. The horizontal line through the boxes illustrate the length of the confidence interval and the boxes show the effect estimates from the single studies

We pooled data from 8175 HIV infected women to estimate the pooled prevalence of HPV infection using meta-analysis. The overall pooled prevalence of all types of HPV infection was 63.0% (Fig. 2) with high heterogeneity across the studies (χ^2^ = 3,782.80 (d.f. = 15); *p* value < 0.001 and I^2^ = 99.6%). The pooled prevalence of high risk HPV was 51.0% (Fig. 3) with heterogeneity of χ^2^ = 1069.84 (d.f. = 12); *p* value < 0.001 and I^2^ = 98.88%. Similarly, the estimated pooled prevalence of low risk HPV was 28.0% (Fig. 4) with heterogeneity of χ^2^ = 296.83; (d.f. = 5); *p* value < 0.001 and I^2^ = 98.32%.

The pooled prevalence of high risk HPV genotypes was also estimated in the studies (i.e. HPV genotypes 16, 18, 26, 31, 33, 35, 39, 45, 51, 52, 53, 56, 58, 59, 66, 67, 68, 69, 70, 73, and 82). The highest prevalence was observed for genotype 16 (20%) followed by 18 (15%) and 52 (13%). Almost all genotypes indicated heterogeneity and the highest heterogeneity was observed in genotype 16 (**I**^2^ = 98.53%) followed by 18 (**I**^2^ = 97.18%) and 31 (**I**^2^ = 96.17%) (Table 4). The HPV genotypes 26,67,69, 70, 73 and 82 reported less frequently in the included studies which is difficult to interpret.

**Table 4:** The pooled prevalence of different genotypes of HPV among HIV-infected women in LMICs

HPV genotypes	Random effects pooled %ES (95% CI)	No of studies	χ^2^	DF	*p* value	I^2^ (%)
16	20 (12.0–28-0)	13	814.56	12	< 0.001	98.53
18	15 (10.0–20.0)	12	390.48	11	< 0.001	97.18
26	4 (2.0–5.0)	3	2.12	2	0.35	5.68
31	11 (7.0–14.0)	11	260.91	10	< 0.001	96.17
33	8 (5.0–11.0)	8	72.55	7	< 0.001	90.35
35	10 (6.0–14.0)	9	120.78	8	< 0.001	93.38
39	5 (3.0–8.0)	7	53.68	6	< 0.001	88.82
45	7 (5.0–10.0)	10	114.13	9	< 0.001	92.11
51	8 (5.0–10.0)	9	37.74	8	< 0.001	78.80
52	13 (9.0–18.0)	9	157.30	8	< 0.001	94.91
53	10 (5.0–16.0)	7	83.80	6	< 0.001	92.84
56	6 (4.0–9.0)	7	45.42	6	< 0.001	86.79
58	11 (8.0–14.0)	11	94.54	10	< 0.001	89.42
59	5 (3.0–7.0)	7	66.49	6	< 0.001	90.98
66	8 (3.0–12.0)	5	44.36	4	< 0.001	90.98
67	2 (1.0–3.0)	2		1		
68	6 (3.0–10.0)	6	74.28	5	< 0.001	93.27
69	4 (2.0–5.0)	2		1		
70		1				
73	5 (2.0–8.0)	4	12.73	3	0.01	76.42
82	5 (3.0–6.0)	4	3.54	3	0.32	15.21

HPV, human papillomavirus; χ^2^, heterogeinity chi-square; DF, degree of freedom; I^2^, heterogeneity; ES, effect size, CI, confidence interval

### Subgroup analysis

**Fig. 5:**
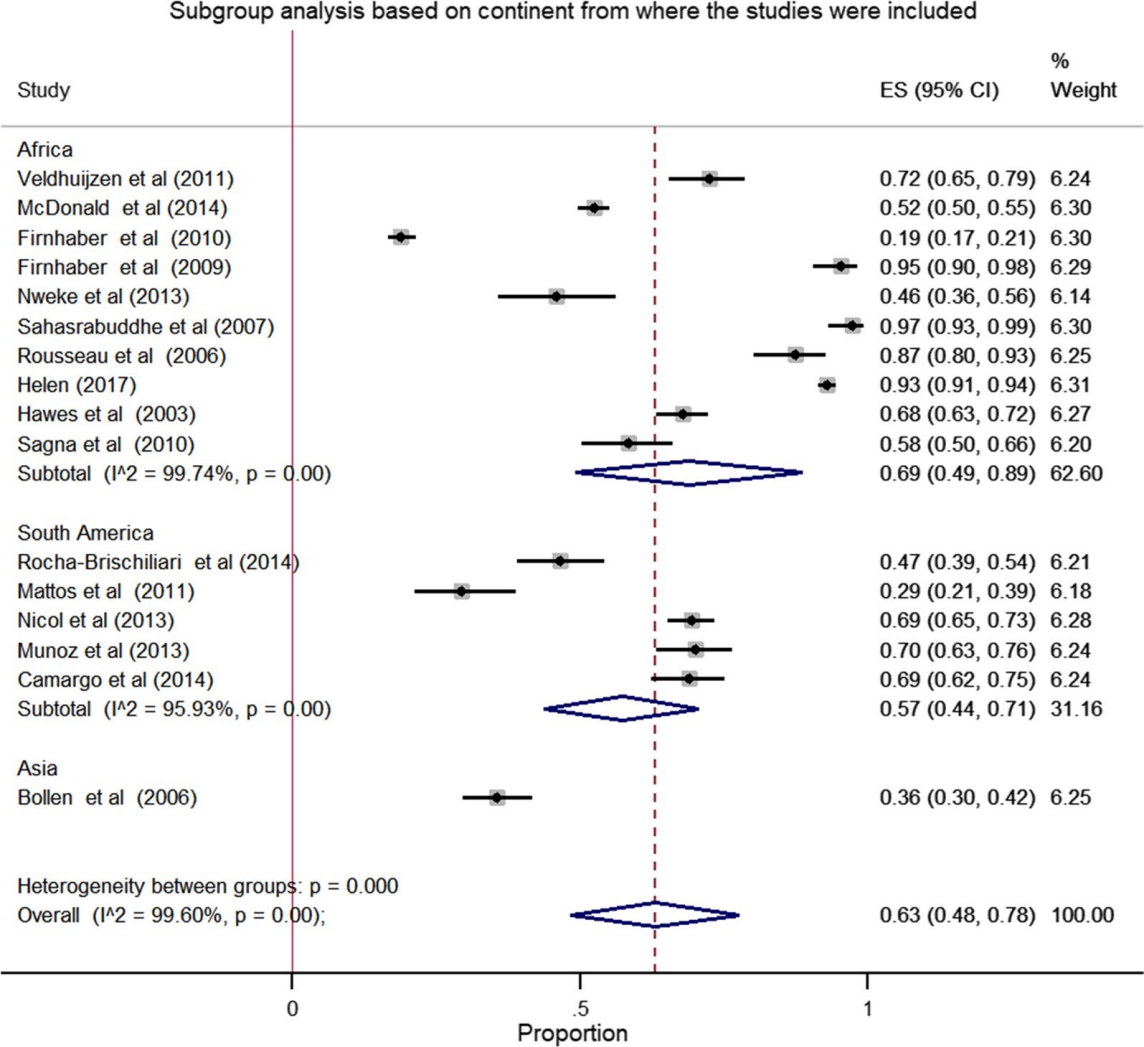
Forest plot of the subgroup analysis based on continent from where the studies were reported. In this plot, three diamond shapes are observed. The first two indicates subtotal prevalence's and the third one indicates the pooled estimate of the prevalence of HPV

The result of subgroup analysis based on the continent from where the studies were include shows significant heterogeneity between and within the group. The pooled prevalence of HPV in African was 69.0% (95% CI: 49.0–89.0) with heterogeneity of **I**^2^ = 99.74% and *p* value < 0.001. The pooled prevalence of HPV in South America was 57.0% (95% CI: 44.0–71.0) with heterogeneity of **I**^2^ = 95.93% and *p* value < 0.001) (Fig. 5).

### Meta-regression analysis

We assessed the effects of sample size and year of the study on heterogeneity between the studies using meta-regression model. Both sample size and publication years significantly predicted the heterogeneity of the effect sizes (Table 5). In the adjusted model, both the sample size and publication year indicated heterogeneity in the effect size which is equivalent to the prevalence (p < 0.001). When we interpret the finding using β-coefficient, one unit increase in the sample size increases the effect size or the outcome of 1.04 points and the outcome decreases by 11.8 points for every one unit increase in the publication year (Table 5).

**Table 5:** Meta-regression analyses for year of study and sample size as a reason of heterogeneity on the prevalence of HPV among HIV-infected women in LMICs

Variable	Adjusted model		
	ß (95% CI)	SE	*p* value
Sample size	1.04 (1.0 to 1.09)	.02	< 0.001
Publication year	− 11.8 (− 16.3 to − 7.2)	2.1	< 0.001

SE, standard error; ß, regression coefficient; CI, 95% Confidence interval

### Publication bias

**Fig. 6:**
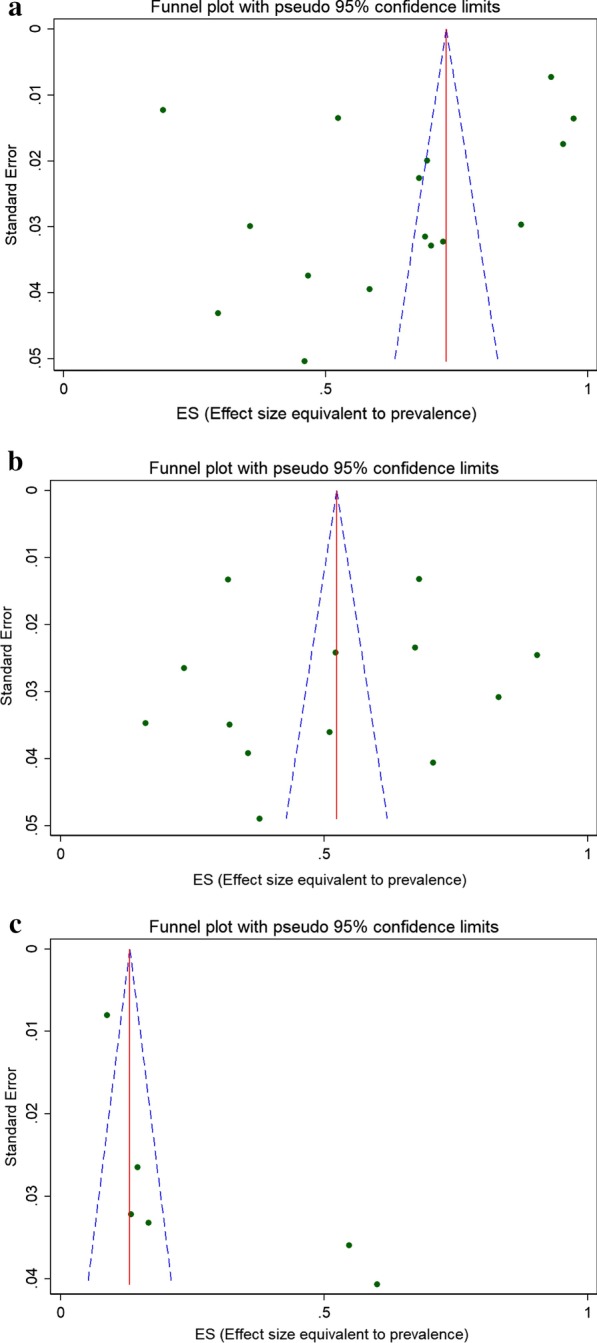
Publication bias assessment: **a** funnel plot of the 16 estimates of HPV types available for meta-analysis (SE—standard error, ES—effect size: prevalence), **b** funnel plot of the 13 estimates of high risk HPV types available for meta-analysis, **c** funnel plot of the 6 estimates of low risk HPV types available for meta-analysis. In this plot, the blue broken line indicates Pseudo 95% CI, the solid red line indicates pooled estimate of the prevalence of HPV, and the scattered circle dots indicates included studies in the meta-analysis. The scale on the X-axis indicates Effect size estimate or proportion and the Y-axis indicates the precision estimate using standard Error

The funnel plot (widely used to examine bias in the results of meta-analysis) for the pooled prevalence of all genotypes HPV, high risk HPV and low risk HPV indicated that there is a publication bias (Fig. 6a–c). Figure 6a, indicates the funnel plot of the 16 estimates of the HPV types available for meta-analysis (SE-Standard error, ES-Effect size: prevalence), (b) Funnel plot of the 13 estimates of high risk HPV types available for meta-analysis (SE-Standard error, ES-Effect size: prevalence), (c) Funnel plot of the 6 estimates of low risk HPV types available for meta-analysis (SE-Standard error, ES-Effect size: prevalence). The majority of included studies were out of 95% confidence limit. The outer dashed lines indicate the triangular region within which 95% of studies are expected to lie in the absence of both biases and heterogeneity. In this funnel plot, scatter properties of the included studies made by medium small size with white background color and the scale of 1.

### Laboratory techniques applied to detect HPV infection in the included studies

Molecular genotyping and HPV detection techniques applied for selected studies were Linear Array HPV Genotyping Test (LA), careHPV, genotyping using PCR-restriction fragment length polymorphism analysis, Reverse line-blot hybridization, INNO-LiPA HPV genotyping Extra® assay (Table 3).

### Factors associated with HPV infection

High HIV viral load and low CD4 count were the most frequently reported factors that associated with high-risk HPV infection [23, 47, 48, 50]. Hormonal contraceptive use, CD4count < 200 cells/mm^3^, history of three or more sexual partners were reported as the factors associated with HPV infection [37, 38] (Table 3).

## Discussion

In the current review, the pooled estimate of HPV infection prevalence was 63.0%. The estimates of high risk and low risk genotypes were 51.0% and 28.0%, respectively. Of high risk genotypes, HPV genotype 16 was high (20%) followed by 18 (15%) and 52 (13%), respectively. Low CD4count and high HIV viral load were the risk factors that most frequently reported in this review.

This finding was lower than the findings in Kenyan which reported 68.0% overall pooled prevalence of high risk HPV among HIV positive women [27]. Genotype 16 was the most prevalent HPV genotype (20.0%) in our review. This finding, however, was different from previous review which reported HPV 52 with pooled estimate prevalence of 26% among HIV infected women with normal cytology and HPV 16 which was 26% among women with abnormal cytology [27]. This difference is likely to be due to the number of included studies and the difference in the data included in the analysis, study setting and participants exposure to risk factors including HIV.

Lower CD4 count most frequently reported in this review is concordant with previous reviews in which low CD4 was strongly associated with HPV infection [53–55]. Previous study also revealed that the most frequent high risk genotype observed in HIV positive women (i.e. 46.7%) [54] which is closer to the current pooled estimate for high risk HPV (51.0%), indicating that HIV infection might increase the susceptibility to latent HPV infection [55].

The review conducted to estimate prevalence of HPV genotype among African women, including Ethiopia revealed that HPV16, 52, 35, 18, 58, 51, 45, 31, 53, and 56 were the ten most common genotypes in the normal cervical cytology while HPV 16, 18, 35, and 52 were the four common types [56]. Another review from Ethiopian women depicted that HPV 16, 52, 18, 58, 45 were top five genotypes with the proportion of 45.3%, 9.4%, 8.2%, 6.9%, 5.2%, respectively [57]. Correspondingly, the pooled estimates of about 21 high risk HPV genotypes among HIV infected women were reported in this review with the estimated prevalence of genotype 16 (20%), 18 (15%), 52 (13%), 31 and 58 (11% each), 35 and 53 (10% each), 33, 51, and 66 (8% each), 45 (7%), and the remaining genotypes had the pooled estimate of less than seven percent.

The original research article conducted in Korea reported prevalence of 16.7% with the high risk HPV type of 12.5% [58] which is too far up when compared with the pooled estimates of the current review which focused on HIV positive women. In addition, the study among Arab women indicated 6.2% among Qatari women and 5.9% non-Qatari women [59] somewhat concordant with the study conducted in Lebanon which reported HPV prevalence of 6.7% [60]. This variation is probably attributed to the differences in the study settings, sample sizes used and the studied population.

Our finding indicated heterogeneity on the outcome variable which is the effect size equivalent to the prevalence of HPV genotypes. Therefore, careful interpretation of the heterogeneity chi-square test (variation in effect estimates beyond chance) is required, since it has low power in the situation of a meta-analysis when studies have a small sample size or are few in number. It is worth noting at this junction that while a statistically significant result may indicate a problem with heterogeneity, a non-significant result must not be taken as evidence of no heterogeneity.

### Strength and limitations of the study

This review is conducted by searching more than five biomedical databases and a large number of pooled participants are involved in the study. Another strength is that this review assessed HPV prevalence studies among HIV infected women in developing countries at large and reported pooled estimates of all HPV genotypes, high risk HPV genotype and low risk HPV. The limitation of this study was inclusion of studies published only in English language. This could be one of the possible causes for observed publication bias and heterogeneity of the estimated effects.

## Conclusion

This review indicated that the pooled prevalence of all genotypes HPV and high risk HPV among HIV infected women in LMICs were considerable. To enhance the well-being of HPV/HIV co-infected women it is necessary to strengthen programs for diagnosis, treatment, and provide HPV vaccination based on common high-risk genotypes.

## Data Availability

All data generated or analyzed during this review are included in this article.

## Abbreviations

CI: Confidence interval
df: Degree of freedom
DNA: Deoxyribonucleic acid
ELISA: Enzyme linked immuno-sorbant assay
ES: Effect size
HAART: Highly active anti retroviral therapy
HIV: Human Immunodeficiency virus
HPV: Human papillomavirus
HSILs: High grade squamous intraepithelial lesions
I^2^: Heterogeneity
ICC: Invasive cervical cancer
LMICs: Low and middle income countries
MeSH: Medical subject heading
PCR: Polymerase chain reaction
SILs: Squamous intraepithelial lesions
STI: Sexually transmitted infection
tiab: Title and abstract
VLP: Virus like particle
